# ECG, LVH and T1 changes in Fabry disease - implications for screening and understanding of the disease model

**DOI:** 10.1186/1532-429X-18-S1-Q48

**Published:** 2016-01-27

**Authors:** Rebecca Kozor, Sabrina Nordin, Amna Abdel-Gadir, Heerajnarain Bulluck, Thomas A Treibel, Charlotte Manisty, James C Moon

**Affiliations:** Cardiology, Barts Heart Centre, London, United Kingdom

## Background

Fabry disease is a rare X-linked condition that results in storage of sphingolipids in multiple organs including the heart. The cardiac phenotype consists mainly of conduction abnormalities, left ventricular hypertrophy (LVH) and disease progression (fibrosis, arrhythmias and heart failure).

CMR LGE classically shows basal inferolateral LGE. Native T1 mapping has recently shown a low T1 likely to represent myocycte storage of fat, even when no LVH is present. We sought to understand the relationship of storage to LVH and ECG abnormalities.

## Methods

67 genetically confirmed Fabry disease patients underwent CMR (function, T1 mapping, LGE) on a 1.5T Siemens Avanto with a concurrent 12 lead ECG. LVH was defined by mass index (LVM_i_) and a wall thickness ≥12 mm. Native T1 mapping used ShMOLLI with a region of interest drawn in the septum in a basal short axis view, and 900 ms considered the lower limit of normal. An abnormal ECG was defined by a prolonged PR interval (>200 ms), prolonged QRS duration (>120 ms), the presence of T-wave inversions, and/or LVH by Sokolow criteria.

## Results

The 67 Fabry patients had a mean age of 46 ± 14 years, females 70% (47/67), and good LV function (LVEF 76 ± 7%). LVH was present in 43% (29/67) by wall thickness and 48% (32/67) by mass index.

**T1 with LVH:** 69% (46/67) had low native T1 values. Low T1 was more common in LVH positive than LVH negative subjects (86% vs 55%, mean 854 ± 53 vs 897 ± 46 ms, p = 0.001). However, 55% (21/38) of the LVH-negative subjects had low T1 values, and 14% (4/29) of LVH-positive subjects had a normal T1.

**T1 with ECG:** 64% (43/67) had abnormal ECGs, (Table 1; results stratified by T1 and LVH). ECG abnormalities were highly correlated to LVH by wall thickness (13 ± 9 vs 9 ± 2 mm, p < 0.001) and LVM_i_ (98 ± 36 vs 67 ± 22 g/m2, p = 0.001) with lower T1 values in those with ECG abnormalities (863 ± 52 vs 906 ± 44 ms, p = 0.001).

**Table 1:** Differences in ECG changes between Fabry patients with and without low T1 values

	Low T1	Normal T1	p-wave	LVH-positive	LVH-negative	p-wave
P-wave duration (ms)	98 ± 20	85 ± 24	0.02	101 ± 22	89 ± 21	0.03
PR interval (ms)	152 ± 31	158 ± 37	NS	156 ± 29	153 ± 36	NS
P-wave to PR interval ratio (%)	67 ± 17	55 ± 12	0.004	67 ± 20	59 ± 13	0.05
QRS duration (ms)	98 ± 24	86 ± 11	0.03	109 ± 24	83 ± 9	<0.001
T-wave inversions (n)	25	7	NS	22	10	<0.001
Sokolow (mm)	33 ± 16	29 ± 10	NS	33 ± 14	31 ± 14	NS

In subjects without LVH, when the T1 was low, 12/21(58%) had an abnormal ECG, and 6/17 (33%) when the T1 was normal.

## Conclusions

In gene positive Fabry disease subjects, LVH has a low T1 85% of the time, but half of all LVH-negative patients have a low T1. When there is no LVH, ECG abnormalities are slightly more common in those with a low T1 - but up to 1 in 3 have ECG abnormalities before apparent storage. The most plausible explanations for this are either that T1 can miss some storage, or that there may be other Fabry effects other than storage - (toxic metabolites are proposed).

**Figure 1:**
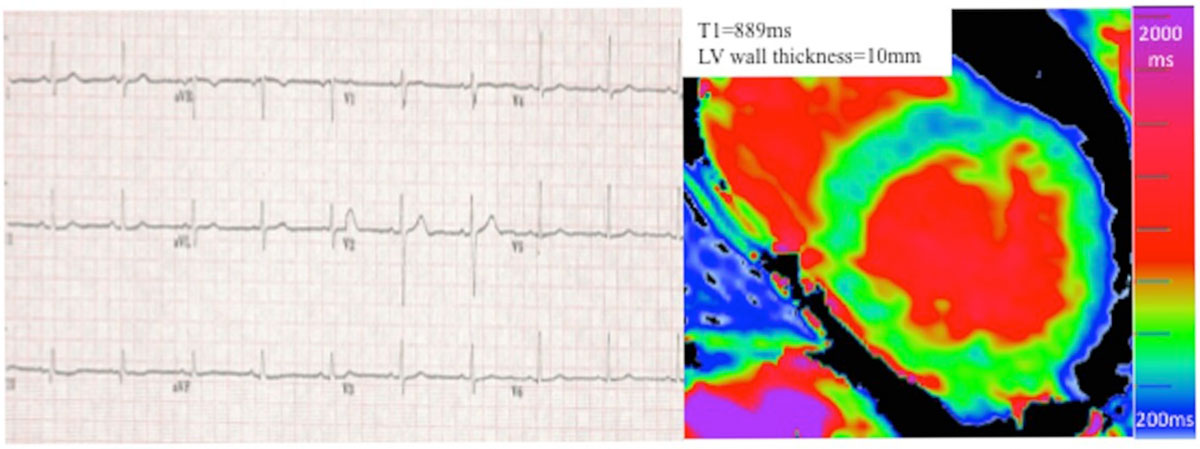
**Normal ECG in Fabry disease patient without left ventricular hypertrophy (wall thickness of 10 mm) but low T1 of 889 ms**.

